# Measuring Spin⋅⋅⋅Spin Interactions between Heterospins in a Hybrid [2]Rotaxane

**DOI:** 10.1002/anie.201612249

**Published:** 2017-03-09

**Authors:** Marie‐Emmanuelle Boulon, Antonio Fernandez, Eufemio Moreno Pineda, Nicholas F. Chilton, Grigore Timco, Alistair J. Fielding, Richard E. P. Winpenny

**Affiliations:** ^1^ The School of Chemistry and Photon Science InstituteThe University of ManchesterOxford RoadManchesterM13 9PLUK

**Keywords:** heterometallic rings, molecular magnetism, pulsed EPR spectroscopy, quantum computing, rotaxanes

## Abstract

Use of molecular electron spins as qubits for quantum computing will depend on the ability to produce molecules with weak but measurable interactions between the qubits. Here we demonstrate use of pulsed EPR spectroscopy to measure the interaction between two inequivalent spins in a hybrid rotaxane molecule.

Molecular electron spins are potential qubits for quantum information processing (QIP).^1^ Much recent work has been dedicated to showing that the phase memory times of *S*=1/2 molecules can be controlled, and extended to the point where multiple spin manipulations will be possible.^2^ One of the key next steps is to study the interactions between such spins. Recently, Ardavan et al. have shown using double electron‐electron resonance (DEER) spectroscopy that the interaction between electron spins in {Cr_7_Ni} rings can be controlled to fall within the range needed for two qubit gates.^3^ These studies were performed on dimers of {Cr_7_Ni} rings^4^ where the *S*=1/2 spins in each half are identical.

Systems containing different spins also have great potential for QIP as there is then the possibility of manipulating each spin separately. This underpins the *g*‐engineering idea proposed by Takui and co‐workers for organic radicals^5^ and work employing heterometallic lanthanide dimers.^6^ Large differences in *g*‐values could also be used as a means to implement entangling two qubit gates.^7^ Quantifying weak interactions between very different spins is challenging. In cases where the interaction can easily be measured, for example by magnetometry or continuous‐wave (CW) electron paramagnetic resonance (EPR) spectroscopy,^8^ the interaction will be too large to permit implementation of both one‐ and two‐qubit gates in the same molecule.^3^ CW EPR spectroscopy can still be a useful tool to investigate dipolar interactions; by observing the line broadening of a N triplet in a two‐qubit structure, Zhou et al. showed the existence of a dipolar interaction that they could estimate in conjunction with spin density calculations.^9^ On the other hand, DEER spectroscopy,^10^ while a powerful tool to measure weak interactions by directly manipulating two weakly interacting spins with specific microwave pulses, is limited as the bandwidth of the microwave source must encompass the resonant frequency of both electron spins.

Here we report a two‐qubit assembly comprising an organic radical within the thread of a hybrid [2]rotaxane containing a {Cr_7_Ni} ring: this is a heterospin system where the constituent spins possess vastly different *g*‐values. To quantify the weak interaction between the spins we use “Relaxation Induced Dipolar Modulation” (RIDME) spectroscopy,^11^ which has been developed in structural biology to measure distances between dissimilar spins.^12^

To make the [2]rotaxane an organic thread was synthesized containing a secondary amine and terminating in an aldehyde (see the Supporting Information for details). Reaction of this thread with hydrated chromium trifluoride and basic nickel carbonate in pivalic acid gave {[Ph‐(CH_2_)_2_‐NH_2_‐CH_2_‐(C_6_H_4_)_2_‐CHO][Cr_7_NiF_8_(O_2_C^t^Bu)_16_]}. A 2,2,6,6‐tetramethyl‐1‐piperidinyloxy (TEMPO) radical was then introduced at the aldehyde group through a Schiff‐base condensation giving {[Ph‐(CH_2_)_2_‐NH_2_‐CH_2_‐(C_6_H_4_)_2_‐TEMPO][Cr_7_NiF_8_(O_2_C^*t*^Bu)_16_]} (**1**). The structure of **1** contains an octagonal {Cr_7_Ni} ring with the metal ions at the corners, with each edge bridged by a single fluoride and two pivalates (Figure 1). The divalent Ni ion is disordered around the eight sites of the octagon. The distance between the oxygen atom of the TEMPO group (which bears the majority of the nitroxide spin density^13^) and the metal ions of the ring varies from 17.07 to 18.14 Å, while the distance to the amine nitrogen atom around which the ring is templated is 16.97 Å. The ring is approximately perpendicular to the thread. While the thread is rather rigid, the N−C linkage between the TEMPO group and the adjacent phenyl ring places the O‐atom off the thread axis; free rotation about this bond would produce a circle with a radius of approximately 2.59 Å. The distance between the radical and the ring is too great for any interaction to be observed by magnetometry or by CW EPR spectroscopy (see Figure S2).

**Figure 1:**
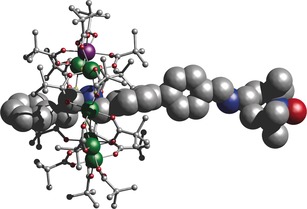
The structure of **1** in the crystal. The ring is shown as a ball‐and‐stick representation and the thread as a space‐filling model. Color coding: Cr dark green, Ni purple, O red, C grey, N blue, F light green. H are not represented for clarity.

The field‐swept echo‐detected (FSED) EPR spectrum of **1** at Q‐band and 5 K shows two well‐separated transitions: a sharp resonance split by ^14^N‐hyperfine centered at ca. *g*=2.007 for the TEMPO radical and a much broader and axial resonance for the {Cr_7_Ni} ring centered at ca. *g*=1.779 (Figure 2); these values are typical for the two spins.^14^, ^15^

**Figure 2:**
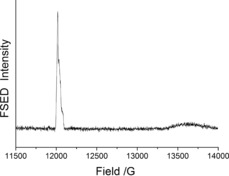
33.823 GHz FSED spectra at 5 K on a frozen toluene solution.

RIDME benefits from the two spins having different longitudinal (spin‐lattice) relaxation times, *T*_1_: in **1** the nitroxide has a very long *T*_1_^n^ (0.2 s at 10 K^16^) and the {Cr_7_Ni} ring has a comparatively short *T*_1_^r^ (ca. 1 μs at 5 K2a). The spin echo of the more slowly relaxing spin is measured and the modulation of this echo caused by the spontaneous flipping of the more rapidly relaxing spin allows the spin⋅⋅⋅spin interaction to be quantified. The RIDME sequence requires pulsed EPR resonances to be measured only at the resonant frequency of the slowly relaxing spin which makes it useful for heterospin systems. RIDME is also less orientation selective than DEER and therefore can benefit from more intense signals and spectral simplicity.

The five‐pulse RIDME12a sequence was used at Q‐band (ca. 34 GHz) at 5 K (Figure 3). This sequence is dead‐time‐free and uses only one frequency centered on the observer spin (the nitroxide, Figure 2). First, a Carr–Purcell Method A sequence (π/2−*τ*−π) tips and re‐focuses the spins in the *xy* plane. The third pulse, after variable time *t*, converts this refocusing transverse magnetization into longitudinal magnetization. The system is then allowed to evolve freely for a time *T* on the order of the longitudinal relaxation time *T*_1_^r^ of the ring (Figure 3). During this time *T*, the ring spin will flip spontaneously with a probability of 1/2 {1−exp(−*T*/*T*_1_^r^)}.^11^ A final refocusing π/2 and π pulse series is used to obtain a refocused virtual echo (RVE). The time between the 3rd and 4th pulses (*T*) is held constant and both pulse positions are incremented at constant rate. The echo intensity is detected as a function of *t* (Figure 3). If the ring spin flips during the evolution time *T*, the resonance frequency of the nitroxide shifts by the interaction frequency, thus modulating the final echo amplitude. The interval time *T* used was 4000 ns, which is around four times *T*_1_^r^ under these conditions (Figure S5). Measurement at Q‐band suppresses electron spin echo envelope modulation effects under these conditions (Figure S6).

**Figure 3:**
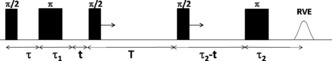
5‐pulse RIDME sequence. The π/2 and π pulses had durations of 20 and 40 ns, respectively, separated by *τ*=140 ns. The positions of the third and fourth pulses were incremented by 4 ns. The experiment was carried out at the nitroxide absorption peak at Q‐band.

Removing a background function of the form exp(−*kt*) where *k*=1.8×10^5^ ns^−1^ gives the experimental form factor that displays a modulation depth of 0.287 (Figure 4 a, raw data is given in Figure S7). Fourier transform gives the frequency–space spectrum which contains two peaks; the separation of these two peaks is a direct measure of 2 *D*, *D* being the magnetic interaction which is around 8 MHz (Figure 4 b). Fitting the data with DeerAnalysis^17^ using a point dipole model based on two localized *S*=1/2 species is wholly inadequate due to the spin density distribution in the ground state of the {Cr_7_Ni} ring.^18^, ^19^

**Figure 4:**
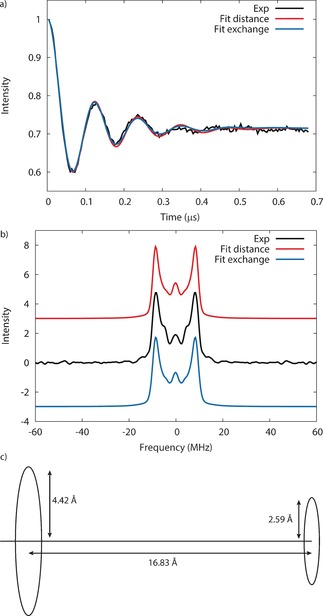
a) Experimental (black) and simulated (red and blue) RIDME traces measured on **1** at 33.8159 GHz and 1.2027 T as a frozen solution in toluene at 5 K. The time axis is “*t*” in Figure 3, which was incremented in 4 ns steps. Red traces use *k*=1.77×10^5^ ns^−1^, mod. depth=0.287, *R*_ring–nitroxide_=16.73 Å, *σ*=0.506 Å and *J*=0 MHz and blue traces use *k*=1.83×10^5^ ns^−1^, mod. depth=0.288, *R*_ring‐nitroxide_=16.83 Å, *σ*=0.607 Å and *J*=+0.15 MHz. b) The Fourier transform of the RIDME traces (experimental, black; simulation, red and blue). c) The simple model used to fit the RIDME data; the large circle represents the spin distribution of the {Cr_7_Ni} ring and the small circle the potential conical motion of the TEMPO radical.

To go beyond this limitation we have simulated the data using an in‐house code SSD (“Spatial Spin Density”), which calculates the form factor directly using a spatially distributed dipolar model^20^ considering the perturbation from the flipping spin of the ring during the time interval *T*. The spin projection coefficients for the *S*=1/2 ground state of {Cr_7_Ni} were obtained using the “Irreducible Tensor Operator” (ITO) technique with the PHI^21^ program. This includes the full microscopic Hamiltonian,^15^ and reproduces the spin distribution measured by NMR spectroscopy.^19^ Using the structure and including random rotations of the TEMPO and ring about the thread (Figure 4 c), we directly fit the RIDME form factor by introducing the standard deviation for a Gaussian distribution of the ring–nitroxide distance, *σ*.

While this one‐parameter model can give a good fit to the data (Figure S8), a significantly better fit (Figures 4 a and b) is obtained when allowing for an isotropic exchange parameter (*J*=+0.15 MHz) or a small change in the average ring–nitroxide distance (ca. −0.1 Å). Both of these models give excellent simulations of the data, revealing that the dipolar interactions perpendicular and parallel to the thread axis are *D*_perp_=9(2) MHz (0.0003 cm^−1^) and *D*_para_=−18(3) MHz (0.0006 cm^−1^), respectively. In previous work where we looked at the interaction between a ring and a single Cu^II^ center^8^ an exchange interaction of around 0.02 cm^−1^ was observed and was unambiguously not dipolar; in this case the exchange parameter is much weaker, ca. 0.000005 cm^−1^.

This magnetic interaction would give a gate time of 125 ns, which falls in the correct range to implement a two‐qubit gate with **1**.^3^ Unfortunately, while RIDME is very good at measuring the interaction between different spins it only addresses one of the two spins. Therefore, if heterospin systems were to be used in quantum algorithms there remains a need for EPR spectrometers capable of pulsing at two distinct frequencies.

## Experimental Section

The crystallographic data of **1** were recorded on a Bruker Prospector CCD diffractometer with Cu_Kα_ radiation (*λ*=1.5418 Å). The structure was solved by direct methods and refined against *F* ^2^ using SHELXTL. CCDC 1502519 contains the supplementary crystallographic data for this paper. These data can be obtained free of charge from The Cambridge Crystallographic Data Centre. Pulsed EPR spectroscopy was measured on 0.002 mm solutions of **1** compound in anhydrous toluene. Pulsed Q‐band EPR measurements (Inversion recovery, ESEEM and RIDME traces) were recorded using an E580 Bruker spectrometer (3 W) equipped with a Spinjet AWG and 2 mm dielectric resonator. Experimental details are found in the Supporting Information.

## Conflict of interest

The authors declare no conflict of interest.

## Supporting information

As a service to our authors and readers, this journal provides supporting information supplied by the authors. Such materials are peer reviewed and may be re‐organized for online delivery, but are not copy‐edited or typeset. Technical support issues arising from supporting information (other than missing files) should be addressed to the authors.

Supplementary
