# Supplementary material for: Measuring Spin⋅⋅⋅Spin Interactions between Heterospins in a Hybrid [2]Rotaxane
Source: Angew Chem Int Ed Engl. 2017 Mar 9;56(14):3876–9. doi: 10.1002/anie.201612249 (PMC5434811; doi:10.1002/anie.201612249)
Supplement: Supplementary file 1 — Supplementary [file ANIE-56-3876-s001.pdf]

Angewandte Chemie
Eine Zeitschrift der Gesellschaft Deutscher Chemiker

Supporting Information

# Measuring Spin···Spin Interactions between Heterospins in a Hybrid [2]Rotaxane

*Marie-Emmanuelle Boulon, Antonio Fernandez, Eufemio Moreno Pineda, Nicholas F. Chilton, Grigore Timco, Alistair J. Fielding,* and Richard E. P. Winpenny**

anie_201612249_sm_miscellaneous_information.pdf

## Table of Contents

I. Experimental Details for synthesis ..... 2

II. Synthetic methods ..... 2

III. Crystallography ..... 4

IV. Experimental Details for EPR experiments ..... 4

V. Continuous-wave EPR spectroscopy and FSED ..... 5

VI. Pulsed EPR experiments ..... 6

VII. Fitting method ..... 7

## I. Experimental Details for synthesis

Unless stated otherwise, all reagents and solvents were used without further purification. The syntheses of the hybrid organic-inorganic rotaxanes were carried out in Erlenmeyer Teflon® FEP flasks supplied by Fisher.

Column chromatography was carried out using Silica 60 A (particle size 35-70 μm, Fisher, UK) as the stationary phase, and TLC was performed on precoated silica gel plates (0.25 mm thick, 60 F254, Merck, Germany) and observed under UV light.

NMR spectra were recorded on Bruker AV 400, and Bruker DMX 500 instruments. Chemical shifts are reported in parts per million (ppm) from low to high frequency and referenced to the residual solvent resonance.

ESI mass spectrometry and microanalysis were carried out by the services at The University of Manchester.

## II. Synthetic methods

### 1- Synthesis of *N-(4-bromobenzyl)-2-phenylethan-1-amine*L1

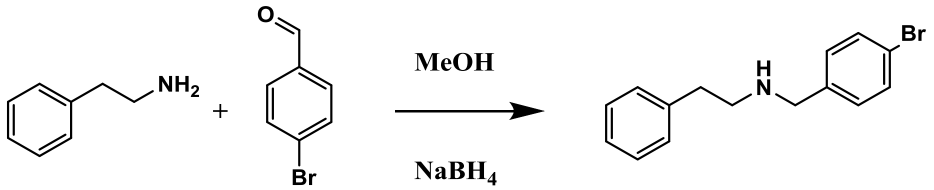


*N-(4-bromobenzyl)-2-phenylethan-1-amine**L1***: To a solution of (4-bromophenyl)methanamine (2 g, 10.7mmol) in 30 mL of methanol, phenethylamine (1.36 mL, 10.7 mmol) in 5 mL methanol was added and the reaction mixture was refluxed for 3 h under nitrogen atmosphere, allowed to stir at room temperature overnight. $NaBH_4$ (5 equiv) was added and reaction mixture was stirred during 12 h under nitrogen atmosphere. The reaction was quenched with water and the solvent was evaporated. The solid was extracted with chloroform, washed with water and dried over anhydrous magnesium sulfate and evaporated. The product***L1*** was obtained as light yellow oil in 72 % yield (2.4 g). ESI-MS (sample dissolved in MeOH, run in MeOH): *m/z* = 290 $[M+H]^+$. $^1$H-NMR (400 MHz, 293K, $CDCl_3$): δ = 2.84-2.91 (m, 4H), 3.85 (s, 2H), 7.00-7.25 (m 7H), 7.81 (d, 2H).

### 2- Synthesis of *4'-((phenethylamino)methyl)-[1,1'-biphenyl]-4-carbaldehyde*L2

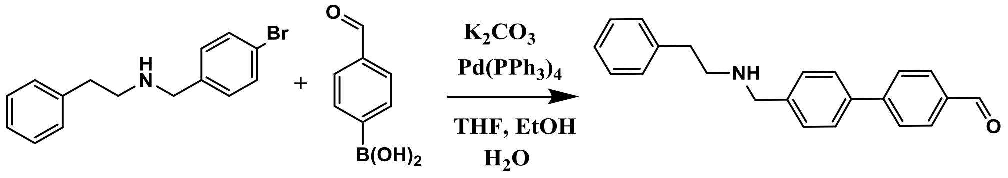


4'-((phenethylamino)methyl)-[1,1'-biphenyl]-4-carbaldehyde***L2***: To a solution of ***L1*** (1 g, 3.4 mmol) in 40 mL of tetrahydrofuran, 12 mL of ethanol and 8 mL of water, 4-formylphenylboronic acid (0.56 g, 3.7 mmol) and $K_2CO_3$ (0.9 g) were added and the solution was purged with $N_2$ for 15 min. $Pd(PPh_3)_4$ (10 % molar) was added to the flask and the mixture heated for 18 hours under nitrogen atmosphere. The solvent mixture was evaporated. The solid was redissolved in chloroform (40 mL), washed with water (2x50 mL) and dried over anhydrous magnesium sulphate and evaporated. A white solid***L2*** was obtained in 70 % yield (0.74 g). ESI-MS (sample dissolved in MeOH, run in MeOH):

m/z = 316 [M+H]$^+$ . $^1$H NMR (400 MHz, 293K, $CDCl_3$): δ = 2.84-2.91 (m, 4H), 3.85 (s, 2H), 7.00-7.25 (m 7H), 7.32 (d, 2H), 7.51 (d, 2H), 7.72 (d, 2H), 7.84 (d, 2H), 9.92 (s, 1H).$^{13}$C NMR (75 MHz, 293 K, $CDCl_3$): δ = 37.8; 46.9; 51.3; 122.8; 125.4; 125.6; 126.0; 127.4; 128.8; 129.7; 129.9; 130.1; 131.5; 133.6; 142.1; 146.4; 151.5; 167.7.

### 3-Synthesis of Rotaxane *P1*

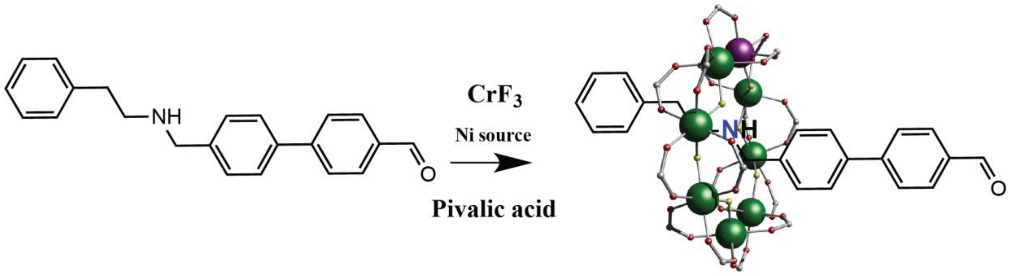


*[2]rotaxane (**P1**)*: Pivalic acid (20.0 g, 195 mmol),***L2***(0.5 g, 1.5 mmol), and $CrF_3 \cdot 4H_2O$ (2.0 g, 11 mmol) were heated at 140 °C with stirring in a Teflon flask for 30 min, then [$Ni_2(\mu\text{-}H_2O)(O_2C^tBu)_4(HO_2C^tBu)_4$] (1.1 g, 2.3 mmol) was added. After 1 h the temperature of the reaction was increased to 160 °C for 20 h. The flask was cooled to room temperature, acetonitrile (50 mL) was added while stirring and a green microcrystalline precipitated was collected by filtration, washed with a large quantity of acetonitrile and dried in air. Flash chromatography (toluene, then gradient elution up to 9:1 toluene: EtOAc) afforded desired [2]rotaxane ***P1*** as a green crystalline solid. Yield: 1.1 g (30 %). Elemental analysis (%) calcd. for $C_{102}H_{166}Cr_7F_8NNiO_{33}$: Cr 14.51, Ni 2.34, C 48.83, H 6.59, N 0.55: found: Cr 14.03, Ni 2.11, C49.30, H 7.18, N 0.59. ESI-MS (sample dissolved in THF, run in MeOH): *m*/*z* = 2531 [M+Na]$^+$; 2509 [M+H]$^+$.

### 4-Synthesis of Rotaxane *1*

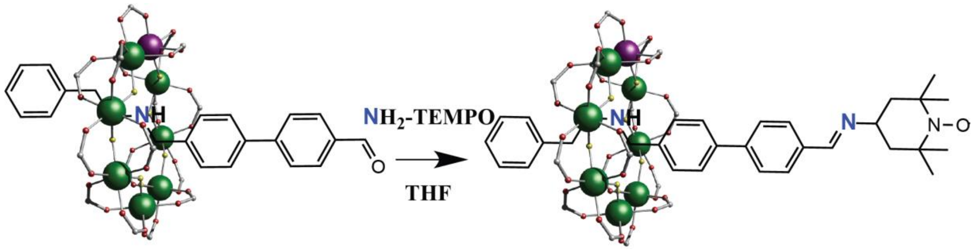


*Rotaxane dimer* (***1***): Amino-TEMPO (0.01 g, 0.05 mmol) was added to a solution of ***P1*** (0.15 g,0.05 mmol) in tetrahydrofuran (10 mL), and the mixture was stirred for 24 h. Tetrahydrofuran was evaporated and the remaining green solid was redissolved in acetone for crystallization. Green crystals, suitable for X-ray structure analysis, slowly formed over a period of several days. The crystalline product was filtered, washed with acetone and dried in air. Yield: 0.1 g (70 %). Elemental analysis (%) calcd for **1**: $C_{111}H_{183}Cr_7F_8N_3NiO_{33}$: Cr 13.67, Ni 2.20, C 50.08, H 6.93, N 1.58; found: Cr 13.51, Ni 2.16, C 49.71, H 6.85, N 1.47. ESI-MS (sample dissolved in MeOH, run in MeOH): m/z = 2663 [M+H]$^+$ .

## III. Crystallography

The data of **1** was recorded on a Bruker Prospector CCD diffractometer with CuKα radiation (λ = 1.5418 Å). The structure was solved by direct methods and refined against $F^2$ using SHELXTL.

CCDC **1502519** contains the supplementary crystallographic data for this paper. These data can be obtained free of charge via www.ccdc.cam.ac.uk/conts/retrieving.html (or from the Cambridge Crystallographic Data Centre, 12 Union Road, Cambridge CB21EZ, UK; fax: (+44)1223-336-033; or deposit@ccdc.cam.ac.uk)

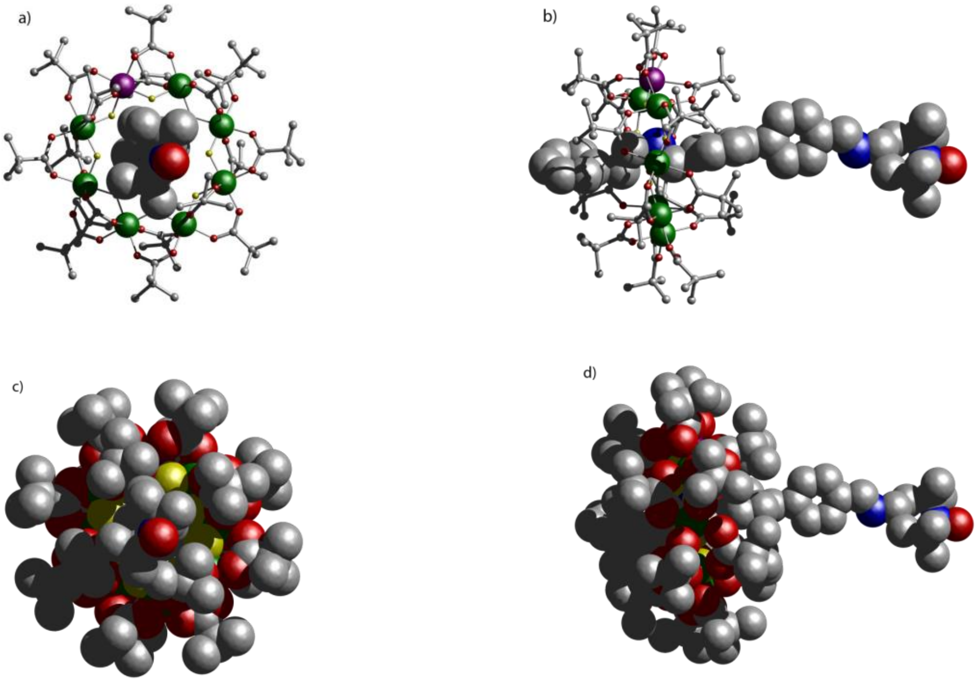


**Figure S1**. Crystal structure of **1** (ball and stick) (a) along the *C*2 axis and (b) side view of the {$Cr_7Ni$} H-atoms omitted for clarity. Space filling representation (c) along the *C*2 axis and (d) side view of the {Cr7Ni}. Colours: Cr, green; Ni, purple; ; F, yellow; O, red; C, grey; N, pale blue.

**Table S1.** Crystallographic information compound **1**

| | **1** |
|---|---|
| Formula | $C_{112.5}H_{184}Cr_6F_8N_3Ni_2O_{33.5}$ |
| Molecular Mass / g mol$^{-1}$ | 2696.04 |
| Crystal System | monoclinic |
| Space Group | $P2_1/m$ |
| $a$ / Å | 16.0010(3) |
| $b$ / Å | 20.7186(3) |
| $c$ / Å | 23.4349(4) |
| $\alpha$ / ° | 90 |
| $\beta$ / ° | 108.775(2) |
| $\gamma$ / ° | 90 |
| $V$ / Å$^3$ | 7355.7(2) |
| $Z$ | 2 |
| $\rho$ calc. / g cm$^{-3}$ | 1.217 |
| $T$ / K | 150(2) |
| $R_1(I>2\sigma)(I))^a$ | 0.0843 |
| $wR_2{}^a$ | 0.2416 |

$^aR_1 = ||F_o| - |F_c||/|F_o|$, $wR_2=[w(|F_o| - |F_c|)^2/w|F_o|^2]^{1/2}$

## IV. Experimental Details for EPR experiments

The C.W. experiment at 5 K (Figure S2) was recorded on a Bruker EMX spectrometer using a super high Q resonator equipped with an ESR900 cryostat. The FSED (Figure S3) was recorded on a E580 Bruker spectrometer using a MD5 dielectric resonator and a CF935 cryostat. These equipment were equipped with an Intelligent Temperature Controller (ITC) Oxford Instruments. The C.W. experiment at room temperature (293 K) (Figure S4) was carried out on a Bruker EMX micro spectrometer using a super high Q rectangular resonator.

The room temperature spectrum was recorded as a mobile solution in dry toline (0.1 $10^{-3-}$ mol $L^{-1}$). All other spectra were recorded frozen solutions in dry toluene at concentrations given in the figure captions. The experiments used 4 mm diameter quartz tubes for X-band and 1.6 mm diameter for Q band spectroscopy.

## V. Continuous-wave EPR spectroscopy and FSED

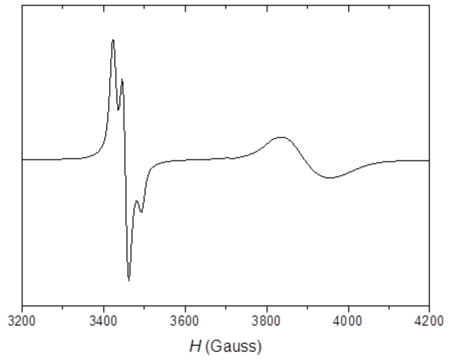


**Figure S2.** CW EPR X-band (9.694 GHz) spectrum of **1** in dry toluene solution (0.2 $10^{-3}$Mol.$L^{-1}$) at T = 5 K.

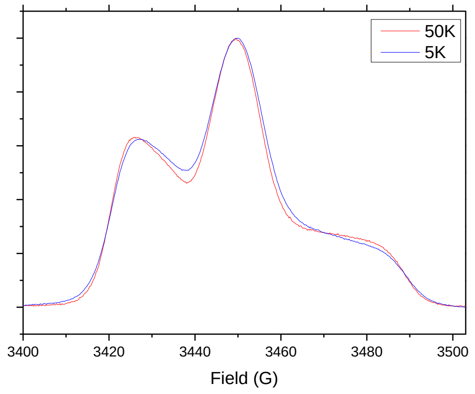


**Figure S3**.FSED spectra of **1** in dry toluene solution (0.2 $10^{-3}$Mol.$L^{-1}$) recorded at 50 K (red) and 5 K (blue). The nitroxide resonance shows broadening due to the dipolar interaction with the $S$ = 1/2 from the {$Cr_7Ni$} ring with $\pi/2$ pulse of 16 ns, $\pi$ of 32 ns and pulse separation $\tau$ = 180 ns.

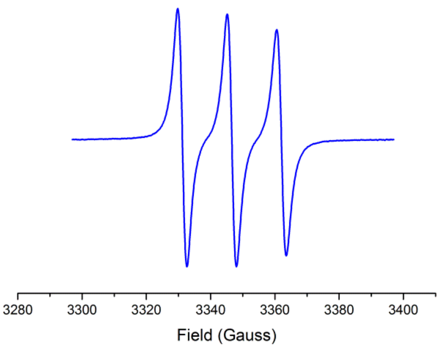


**Figure S4**. CW EPR X band (9.416 GHz) spectrum of **1** recorded at room temperature (*T* = 293 K) in a solution of 0.1 $10^{-3}$Mol.$L^{-1}$in toluene.

## VI. Pulsed EPR experiments

### Inversion recovery

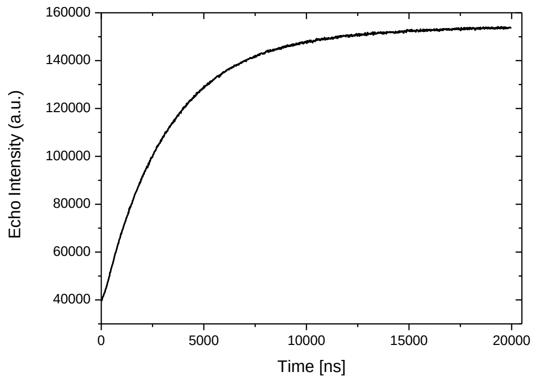


**Figure S5.** Q band inversion recovery at 5 K, 12690.00 G (same sample as RIDME trace, 0.2 $10^{-3}$ Mol.$L^{-1}$ frozen solution of dry toluene) with $\pi$ - $\tau$ - $\pi/2$-$T$-π sequence, $\pi/2$ pulse of 20 ns, $\pi$ of 40 ns and $\tau$ = 140 ns incremented by 4 ns steps.

### 3p ESEEM at 30 K, 12017.00 G

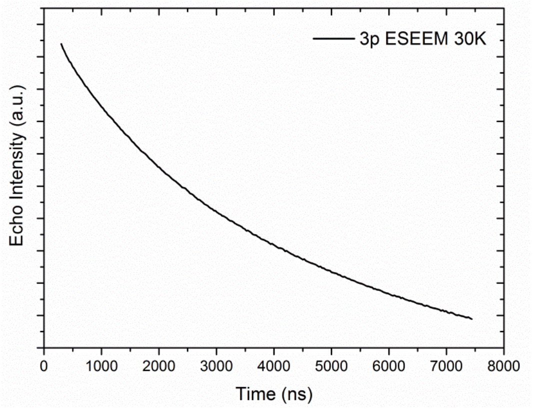


**Figure S6**: Q-band ESEEM recorded at 30 K (same sample as RIDME trace, 0.2 $10^{-3}$Mol.$L^{-1}$frozen solution of dry toluene) measured with a 3 pulse $\pi/2$ - $\tau$ - $\pi/2$ - $\tau_2$- $\pi/2$ sequence, $\pi/2$ pulse of 20 ns.

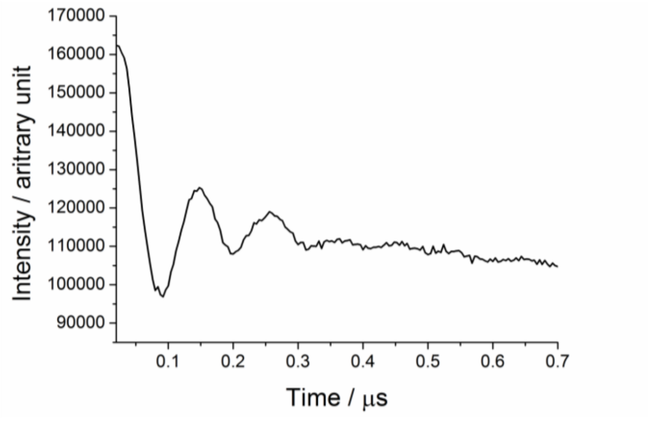


**Figure S7:** Q-band raw data of the RIDME trace recorded at 5 K before background subtraction (Figure 4a in the main text).

## VII. Fitting method

The raw RIDME data was first phase corrected using the average phase difference across the spectrum. Then, the data was background corrected by dividing out a function of the form exp(-*kt*) (see reference 17 of the main text). The RIDME trace was then normalized to the maximum intensity and this time taken as the zero time. This background correction was performed iteratively during the fitting process to find the optimal background parameter and modulation depth. The form factor was calculated following the procedure outlined in reference 20 of the main text, modified to represent the RIDME experiment instead of the DEER experiment. This was done by assuming that the fast-relaxing spin (the ring) flips during the evolution of the nitroxide (all $\lambda_B = 1$ in equation 16 of reference 20), and ignoring detection on the ring (all $f_B = 0$ in equation 16 of reference 20). We integrate over all orientations of the magnetic field with respect to the model geometry, and account for an even distribution of relative ring-nitroxide rotations about the thread axis. The Gaussian distribution of the distance between the ring and the plane of the nitroxide spin is introduced in order to represent structural distributions in solution, and is largely responsible for the decay of the RIDME oscillations within the experimental window.

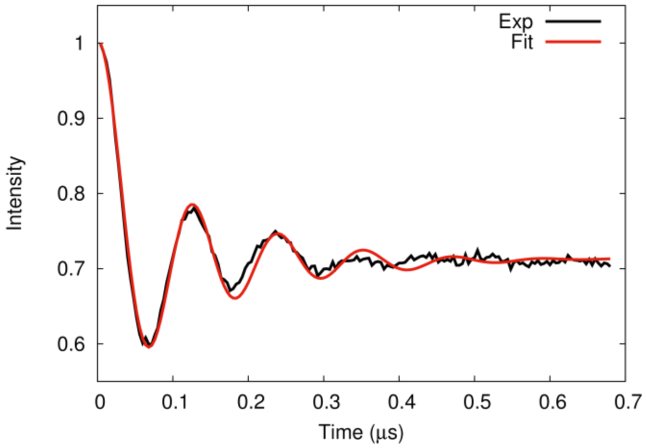


**Figure S8:** Experimental (black) and simulated (red) RIDME traces measured on **1** at 33.815855 GHz and 1.2026960 T as a frozen solution in toluene at 5 K. Fit uses $k = 1.76*10^5$ ns$^{-1}$, mod. depth = 0.289, $R_{ring\text{-}nitroxide}$ = 16.83 Å, $\sigma$ = 0.452 Å and $J$ = 0 MHz.
